# Cascade counselling and testing. Recommendations of the European Society of Human Genetics

**DOI:** 10.1038/s41431-025-01945-3

**Published:** 2025-12-14

**Authors:** Guido de Wert, Carla G. van El, Angus Clarke, Christophe Cordier, Florence Fellmann, Maurizio Genuardi, Sabine Hentze, Hülya Kayserili, Milan Macek, Rhona MacLeod, Béla Melegh, Álvaro Mendes, Emmanuelle Rial-Sebbag, Vigdís Stefánsdóttir, Lisbeth Tranebjærg, Fiona Ulph, Francesca Forzano

**Affiliations:** 1 Department of Health, Ethics and Society, Faculty of Health, Medicine and Life Sciences, CAPHRI Care and Public Health Research Institute, GROW School for Oncology and Reproduction, Maastricht University, Maastricht, The Netherlands; 2 Section Community Genetics, Department of Human Genetics, and Amsterdam Public Health research institute, Amsterdam UMC, Vrije Universiteit Amsterdam, Amsterdam, The Netherlands; 3 Division of Cancer and Genetics, Cardiff University School of Medicine, Cardiff, UK; 4 Sonic Suisse SA, Genesupport, Lausanne, Switzerland; 5 FeelGood Onco, Clarens, Switzerland; 6 Department of Life Sciences and Public Health, Università Cattolica del Sacro Cuore, Rome, Italy; 7 Medical Genetics Unit, Fondazione Policlinico Universitario A. Gemelli IRCCS, Rome, Italy; 8 Synlab, Practice for Human Genetics, Heidelberg, Germany; 9 Koc University School of Medicine (KUSOM) Medical Genetics Department, Istanbul, Turkey; 10 Motol University Hospital—2nd Faculty of Medicine, Charles University, Prague, Czechia; 11 Manchester Centre for Genomic Medicine, Manchester University NHS Foundation Trust, Manchester, UK; 12 University of Pécs, Pécs, Hungary; 13 CGPP—Centre for Predictive and Preventive Genetics, IBMC – Institute for Molecular and Cell Biology, University of Porto, Porto, Portugal; 14 i3S—Institute for Research and Innovation in Health, University of Porto, Porto, Portugal; 15 Department of Diagnostics and Intervention—Oncology, Umeå University, Umeå, Sweden; 16 CERPOP, Bioethics team, Inserm, Université de Toulouse, Toulouse, France; 17 Department of Genetics and Molecular Medicine, Landspitali University Hospital, Reykjavik, Iceland; 18 The Kennedy Centre, Department of Clinical Genetics, University Hospital, Copenhagen, Denmark; 19 Institute of Clinical Medicine, University of Copenhagen, Copenhagen, Denmark; 20 University of Manchester Division of Psychology & Mental Health, School of Health Sci, Manchester, UK; 21 Guy’s and St Thomas’ NHS Foundation Trust, London, UK; 22 Faculty of Life Sciences & Medicine, King’s College London, London, UK

**Keywords:** Medical ethics, Genetic testing, Genetic counselling

## Abstract

Cascade testing (CT) is an effective instrument for identifying an index patient’s relatives at high risk of a heritable condition enabling informed decision-making on preventive interventions and reproductive choice. However, CT remains underutilised and faces barriers. Discussions are ongoing on how to optimise informing family members and testing uptake in a responsible manner. The European Society of Human Genetics (ESHG) contributes to this debate and provides recommendations based on an ethical analysis of when CT is justified, or may be less compelling, considering proportionality and the judicious use of finite resources. ESHG underscores the strong consensus regarding the ‘moral architecture’ of CT in cases of a high risk of serious, avoidable harm. In such cases, a more active approach towards CT is suggested, including a more directive approach in counselling, more active support for the proband, direct contacting, and balancing confidentiality when this is necessary to avoid a high risk of serious harm, taking account of national regulations and jurisdictions. In contrast, more caution is advised in more complex cases where the balance of benefits and harms of CT is less clear, such as when penetrance is low, and actionability or medical treatment is limited. This more cautious approach does not call for directivity, direct contact or the relaxing of medical confidentiality. The focus, then, shifts to cascade counselling, rather than cascade testing. In some cases, CT may not be proportional or appropriate given the balance between benefits and harms, also in view of available resources.

## Introduction

Genetic healthcare has always been focused on understanding the causes and development of genetic disorders and assessing the risk of occurrence or recurrence in individuals and/or their families. The identification of pathogenic variants related to rare genetic disorders has enabled more accurate diagnoses and predictive testing. Guidelines for counselling and testing first-degree family members have been developed to support these efforts [1]. Recent advancements in genetics—most notably regarding cancer predisposition, cardiac disease and neurodevelopmental disorders—have led to increased identification of index patients, allowing for the subsequent informing of at-risk family members.

Cascade testing (CT) is an effective instrument for identifying individuals at high risk for a genetic condition, enabling informed decision-making regarding preventive interventions. It involves a more or less systematic approach to identifying an index patient’s relevant at-risk family members to inform and counsel them about their risk of developing the disease or having an affected child, and offer them targeted testing for the genetic variant found in the index patient. In the absence of finding a pathogenic variant clinical evaluations may be used to assess the risk or health problems for family members.

Identifying carriers of recessive conditions in the broader family enables couples to assess their reproductive risk and, where applicable, access carrier screening for related and unrelated partners. This is particularly important for relatively common conditions for which a genetic test is available but no population screening is offered, as applies to beta-thalassaemia in several Mediterranean and Middle Eastern countries.

There is considerable variation in when and how CT is pursued across and within countries in Europe and globally. Approaches vary both by disorders and their inheritance patterns. Regulations and professional recommendations differ between jurisdictions on whether and how to inform family members [2, 3]. Moreover, a recent systematic review of normative documents on the disclosure of genetic information to relatives suggests there is a lack of clarity regarding the role of healthcare professionals in facilitating that disclosure [4].

CT remains underutilised. In inherited cardiovascular diseases, the uptake of genetic counselling and testing among at-risk family members has been reported to be around 40% [5]. For Lynch syndrome and Hereditary Breast and Ovarian Cancer (HBOC) syndromes variable, but roughly similar percentages have been reported [6–8]. However, it is often difficult to evaluate the reasons why CT has not resulted in the uptake of genetic testing because such testing relies on many different processes and individual considerations. This includes the effective communication by the patient to their relatives about their risks of developing a disease or having affected or at-risk offspring; the adequate understanding of their situation by the family members; their decision to contact their general practitioner to discuss and obtain a referral, or contact genetic services directly; and the knowledge and willingness of the general practitioner regarding referral. In addition, after being referred and counselled, some family members may decide not to undergo testing.

Many studies have set out to examine the reasons for the low uptake of cascade testing. A systematic review clustered barriers and facilitators for CT, differentiating between factors at the individual level (including demographics, knowledge, attitudes, beliefs, and perceptions of relatives) and factors at the interpersonal level (including family communication and support and provider factors). Barriers at the structural level, such as accessibility of genetic services, were also identified [9].

Much attention has been devoted to how family members should be informed about their risk in a responsible manner. Following Newson and Humphries [10], it would be advisable to involve the proband in cascading information to relatives. Patient-mediated approaches may be perceived as enhancing autonomy and privacy and being less disturbing to the relatives [10, 11], but also come with challenges. Some studies suggest that uptake of genetic testing is lower when probands rather than genetic health care professionals inform family members [6, 9, 12, 13]. More active involvement of healthcare professionals is being studied, including directly contacting relatives [14–16]. Discussion is ongoing on how to optimise informing the family in case index patients are unable or unwilling to do so, while respecting the confidentiality of the genetic information and the privacy of the index patient and psychological well-being of family members who are approached without prior notice. In recent years, while balancing the interests of the index patient and family members at risk, more attention has been given to the right of family members to be informed and the option for healthcare professionals to actively initiate family contacts [17–19]. For instance, in the United Kingdom, a recent legal ruling established that healthcare professionals managing a case must consider the potential duty of care to relatives and weigh that against the confidentiality owed to the index case [17, 20]. In the Netherlands, a multidisciplinary guideline has been implemented focusing on offering more support to the index patient in informing family members and, if desirable, granting the medical specialist more leeway to contact family members directly [21]. This change in attitude and guidelines is relevant beyond clinical genetics, as, for instance, oncology, cardiology, internal medicine and other specialties involved in the care of patients with a genetic disease have increasingly become involved in CT. How to organise CT and integrate such evolving standards and expertise in mainstream medicine represents a challenge. Despite these developments, most European countries lack specific regulations about informing patients’ relatives of their genetic risks, making it unclear what duties patients and healthcare professionals have towards at-risk relatives, and what options health care professionals have to assist patients in communicating with their relatives [4, 22].

Another important development potentially affecting the use of CT lies with recent advances in genomic DNA sequencing techniques. CT has been prioritised over e.g. population screening in specific disease groups because of a higher chance of detecting individuals at risk, allowing for an optimal use of resources [23, 24]. In the absence of a medical family history for a disorder, it may be less straightforward to assess the penetrance of pathogenic variants [23, 25]. It is relevant to monitor technological and demographic developments to assess if and when the balance may shift based on expanding knowledge of the penetrance of pathogenic variants in a population or subpopulation and cost-effectiveness studies [26–28].

In this changing landscape, it is relevant to take stock of recent experiences and emerging practices in CT. The European Society of Human Genetics (ESHG) regards it as its professional responsibility to contribute to this ongoing debate. There are circumstances in which CT is justified, but experience tells us that it is still not fulfilling its potential. In response to a specific request from the ESHG Board, this document provides recommendations after an ethical analysis regarding:

Under what circumstances is CT justified, and when is it not justified or when might the case for CT be less compelling, based on its proportionality, the actual helpfulness of CT and the judicious use of finite resources?

The document is structured as follows: the section ‘Conceptual clarification’ outlines the conceptual clarification of the process of CT. The section ‘The moral framework of cascade testing’ sketches the basics of the moral framework of CT. The section ‘Toward a more supportive and active approach in cascade testing: removing barriers’ focuses on some cases that are, in principle, uncontroversial, arguing that a more active CT approach is justified to increase its effectiveness. The section ‘More complex cases require a more cautious approach’ focuses on and guides the handling of some more dilemmatic cases of CT. The section ‘Cascade testing beyond the clinical genetic practice’ explores CT beyond the confines of clinical genetic practice in the context of mainstream care, public health, and screening. The final section provides Recommendations. All sections take account of relevant recommendations or guidelines from ESHG and other professional societies. Clinicians should always consider the relevant regulations in their country.

A writing group from the ESHG’s Public and Professional Policy Committee (PPPC)—renamed the Policy and Ethics Committee (PEC) in 2023 - prepared the draft, which was discussed by PEC and invited experts. It was made available for ESHG membership consultation from 25 June until 1 August 2024. The authors have integrated suggestions where appropriate. The Board of ESHG approved the final version on 23 December 2024. Given rapid developments in the field and the need for further reflection, these recommendations will need regular evaluation and updates in the future.

## Conceptual clarification

### Cascade testing

Thus far, there is no consensus regarding the proper nomenclature and definition of CT. This section focuses on the nomenclature and suggests a working definition that is sufficiently wide to cover the diversity of CT practices. Additionally, it focuses on relevant aspects of the process of CT as a background to the reflection in the next sections.

*Cascade testing or cascade screening?*

While traditionally, the term cascade *screening* was widely used, in recent literature, the term cascade *testing* seems to be considered more appropriate; as an example, see the USA NIH National Cancer Institute Dictionary of Genetics Terms [29]. This lack of semantic and classificatory consensus is not surprising. After all, widely used criteria for screening are two-fold: the offer of the testing at hand is unsolicited, and there is no direct medical indication for such testing. Contacting and informing relatives about familial genetic risk meets the first criterion, but the relatives contacted are indicated for genetic testing, although perhaps unknowingly, given their high a priori genetic risk.

In some cases, for reasons of laboratory efficiency, CT involves obtaining information on more sequence variants/genes, which may or may not be associated with the original condition, such as when using cancer gene panels. However, if the wider search would go beyond such ‘medically targeted’ considerations, it is to be qualified as *screening*, as it would, then, meet both criteria for screening. It could be argued that such screening of other gene variants would fall under the heading of opportunistic genomic screening (OGS). This will be briefly elaborated in section ‘Opportunistic genomic screening in cascade testing’. In this document, we use the term cascade *testing*, while acknowledging that there is a grey zone between testing and screening and that, depending on the strategy used, cascade *testing* may be more similar to *screening*. Awareness of the type and scope of the test used by the laboratory is important to allow for adequate counselling and consent also regarding secondary findings.

### Cascade testing: a process

CT is shorthand for a process or trajectory comprised of different steps, each of which raises organisational, logistic and normative issues:

1. The genetic counselling and testing of the proband with a medical problem.
  Before ordering a germline genetic test, counselling typically involves an early discussion with the adult patient of the risks and implications for relatives, as well as an assessment of issues that may arise in the communication with them.
2. The identification of relevant genetic relatives at a priori high(er) risk, using a family tree/pedigree.
  CT involves a stepwise, systematic approach to identify relatives and inform them about their familial genetic risk, starting with living first-degree relatives, or second-degree relatives when these first-degree relatives are deceased.
3. The engagement of the proband in the process of contacting/informing those relatives, offering pre-test counselling.
  The default procedure is to engage the proband in contacting and informing their family members. This ‘indirect’ or ‘mediated’ contacting (via the proband or another family member) is distinguished from ‘direct’ or ‘active’ contacting (via a professional), and which requires access to the contact details of the proband’s relatives. The ‘direct’ approach may help overcome difficulties in family communication, thereby maximising the number of relatives approached and thus facilitating a more systematic offer of CT, however, national regulations vary on whether and in what way such an approach is allowed, and it involves practical challenges [30].
4. The pre-test counselling of family members.
  Counselling is a separate step in the process of cascade testing, which should be considered independent of testing. As will be argued in this document in some scenarios, cascade counselling (CC) rather than cascade testing should be emphasised.
5. The testing and post-test counselling of family members.
  CC and CT of relatives for a specific genetic risk enables them to take preventive actions, e.g., medical interventions and/or consider their reproductive choice. Quite often, CT is defined in terms of the former. In many cases, the genetic information communicated primarily for medical reasons may also have implications in the reproductive domain and vice versa. Given this, the current document makes a distinction between *medical* CT (primarily for medical reasons: primary prevention, or earlier diagnosis and intervention) and *reproductive* CT (primarily for reproductive reasons)—though, the two may often overlap.
6. Widening the circle of contacts: contacting/informing relatives in the next degree of kinship.

The index person may increasingly be an asymptomatic carrier of a pathogenic (or likely pathogenic) gene variant, due to a finding generated in the context of population screening, or a secondary finding in the context of OGS. In the latter case, CT may be implemented ‘in the slipstream’ of OGS, the justification of which has been stated explicitly to also serve the health interests of relatives [31].

Taking account of the diversity of CT practices, we make use of a wide, inclusive, working definition of CT: after identifying a (likely) pathogenic variant in an index patient, CT involves a systematic approach of identifying relevant family members to inform and counsel them about their own risk of developing the disease or having an affected child and offer them targeted testing for the variant found in the index person. This process is repeated in subsequent degrees of kinship as more carriers are identified in the family.

Please note that CT is considered here a process which should benefit the family members, and it is considered different from the inheritance studies performed in selected cases to aid the interpretation of a variant of uncertain significance (VUS) in the proband. CT for a VUS would not be considered appropriate.

## The moral framework of cascade testing

This section sketches the basics of the moral framework of CT.

As the tenet of clinical genetics is: ‘the patient is the family’, the offer of cascade testing is at the heart of good clinical genetics practice.

### Proportionality: balancing benefits and harms

Proportionality in the strict sense refers to the balance of possible benefits and harms for individuals involved, while proportionality in a wider sense also involves broader (‘societal’) implications. The latter will be addressed under the heading of justice (see below).

CT is implemented because of its expected benefits for relatives of a proband. Such benefits may be rather diverse. Often, CT is being offered primarily for medical reasons in the context of primary prevention, i.e. the prevention (or at least the delay of onset) of one or more specific genetic disorders, or secondary prevention, i.e. early (pre-symptomatic) detection of disease, aimed at improving the prognosis of affected relatives. A subset of CT primarily enables prospective parents to make informed reproductive choices.

Possible harms of CT include adverse consequences for relatives’ psycho-emotional well-being and negative societal repercussions. Concerns linked with relatives’ well-being include that CT generates anxiety in being contacted and informed about their future health, that it may impose a burdensome trajectory of follow-up examinations, periodic monitoring and preventative surgery, and that it confronts them with complex reproductive dilemmas and disapproval of their choices by others. Possible societal harms include repercussions for individual carriers’ access to, for example, life insurance [9] or employment [32]. The magnitude of this risk depends on the adequacy of the protection offered to those tested in different (national) jurisdictions. It would be problematic to refrain from (or to stop) offering CT that provides relatives with clear medical and reproductive benefits because of such possible societal repercussions for carriers thus identified. The better alternative would be to scrutinise and help remove or amend regulations or practices that function as a barrier for otherwise proportional CT [12].

CT should only be implemented if the benefits are clear and evidence-based, the benefits outweigh the harms, and adequate pre- and post-test genetic counselling is provided. While CT may confront family members and prospective parents with difficult (reproductive) dilemmas, many would argue that it is better to be informed about a high a priori avoidable risk than not [33].

To conclude, the offer of CT is justified, or even imperative, if and insofar as there is strong evidence that the benefits of CT outweigh both the disadvantages and the opportunity costs. The real question is not *whether* CT can be justified but *when* and *under what conditions*.

### Respect for relatives’ autonomy

A basic principle of biomedical ethics is respect for autonomy or self-determination. Some people are concerned that the offer of CT undermines respect for relatives’ autonomy in different ways, also depending on the age of the relatives.

#### Adults

Autonomy in this context includes relatives’ self-determination regarding receipt of familial information, genetic testing and possible medical prevention and reproductive choice.

The first concern is that CT may be a violation of relatives’ right to *not* know [34]. It is, however, important to unravel this presumed right, and to distinguish its different layers: a) the right not to be *unsolicitedly* informed about familial genetic risks, b) the right to decide about genetic testing and c) the right, on further consideration, to not be informed about available test results. The current criticism regards the first layer. However, informing relatives about familial genetic risks could be more appropriately considered as an enabling step to implement relatives’ right to decide about genetic testing (layer b). An ethical view that focuses exclusively on relatives’ right to *not* know (esp. layer a) does not do justice to safeguard relatives’ possible health and/or reproductive interests and may even violate relatives’ right *to know* [35].

The second concern is that the offer of CT may undermine autonomous decision-making, not just because individual caregivers may manifest a directive attitude, but also given the ‘structural’ directivity inherent in the offer, as can arise in the different context of antenatal screening [36, 37]. However, this can only be a convincing categorical objection to CT if strict ‘neutrality’ is always and necessarily morally required and directivity is always and necessarily morally flawed/problematic, irrespective of the (medical or reproductive) context, and even if in moderation.

All in all, the offer of CT may well meet the principle of respect for autonomy. The pre-requisite of informed consent is, as always, important. As the proportionality of CT may seem to be self-evident to some caregivers (and relatives), it is tempting to forget that informed consent is best construed as a process and a dialogue, giving sufficient room for relatives’ reflection and a ‘subjective standard of disclosure’ (in addition to the ‘reasonable person’ standard), thereby enabling relatives to bring in their questions and concerns. Likewise, frustration regarding the possible under-use of CT is not a particularly good reason to disregard the requirement for proper informed consent; rather, it is a reason to identify and mitigate relevant barriers to offering CT, whenever such testing is proportional (see section ‘Toward a more supportive and active approach in cascade testing: removing barriers’).

#### Minors

Pre-symptomatic testing for adult-onset disorders should generally be postponed until children reach the age of competence and can make autonomous decisions themselves [38–41]. This restraint also pertains to testing for relatively common genetic disorders such as Lynch syndrome and *BRCA*-related cases of hereditary cancer, as such testing has substantially increased in recent years and lack of guidance may result in unwarranted testing of minors.

The right to informational self-determination (including the right to *not* know) should be accorded to children as well, as a specification of children’s right to ‘an open future’ [41–44] - as far as is reasonably possible. An exception regards predictive testing, which may have, in some specific instances, an imminent medical benefit for the child tested. This view should also guide cascade testing in children. Such testing in children for *early-onset* conditions does not violate children’s right to not know—on the contrary: it can be regarded as good clinical care/practice. Relevant examples include CT e.g. for familial hypercholesterolaemia (FH), as there is strong evidence that statins from age 8 onwards help reduce morbidity [45, 46], for familial adenomatous polyposis (FAP), enabling timely monitoring and surgery, and for multiple endocrine neoplasia (MEN) type 2A, aimed at timely surgery. In such cases, not offering CT to parents or a parental refusal of the offer to timely test their child(ren) would be at odds with the interest of (vulnerable) children to be protected from serious harm and would therefore violate children’s right to know and to adequate care.

### Confidentiality

Clinical guidelines recommend that healthcare professionals encourage their patients to communicate relevant genetic information with their relatives and support them throughout that communication [4, 47]. Sometimes, however, the proband is either unable or unwilling to contact and inform relatives. A difficult situation may then arise, given the principle of medical confidentiality. Different views regarding the sound handling of such situations can be found in the literature.

The first cluster of views states that such situations can and should be prevented from arising. A first ‘preventive’ option would be to make access to genetic testing conditional upon the prior agreement to disclose information to relatives. This policy is, however, both problematic, as it entails a ‘coercive offer’ (i.e. would the consent given be genuinely valid?), and ineffective, as probands may revoke their consent thereby confronting the counsellor with the dilemma that was seemingly prevented. An alternative preventive option, some argue, would be for the healthcare provider to inform relatives only in rather general, non-identifying, terms, thereby avoiding a violation of the patient’s right to confidentiality [48]. Though this strategy may seem effective, it may often, however, be illusory; given the small size of modern families, relatives will regularly be able to infer the identity of the proband.

A second cluster of views denies that a possible refusal of the proband to inform relatives creates a real dilemma. The traditional variant of this position is that medical confidentiality is absolute and a priori exceeds any other value. However, this absolutist stance does not account for other relevant considerations. The other perspective, the so-called ‘joint account’ model, argues that genetic information regarding individuals actually belongs to their ‘broader’ families, which demands the routine sharing (of the benefits) of such information, except in exceptional circumstances [49].

The third view, which is increasingly supported in the international literature on the ethos of clinical/medical genetics, and is endorsed by ESHG, is that current situations require a delicate balancing of relevant values [50–52]. From this view, medical confidentiality, though highly important, is not absolute. And while the welfare of relatives is undoubtedly of great importance, that does not mean that the proband’s genetic information necessarily belongs to the relatives as much as to the proband. General criteria for justifiably overruling medical confidentiality, as formulated by the World Medical Association [50], the UK Royal College of Pathologists [52], and the UK General Medical Council [51], for example, are:

a) reasonable efforts to elicit voluntary consent to disclosure have failed;

b) there is a high probability both that harm will occur if the information is withheld and that the disclosed information will be used to avert the harm;

c) the harm that identifiable individuals would suffer would be serious; and

d) appropriate precautions are taken to ensure that only the genetic information needed for diagnosis and/or treatment of the disease in question is disclosed.

### Justice

In discussions of justice, two distinct components emerge.

Firstly, formal justice requires equal treatment of similar cases, considering the two relevant criteria for cascade testing: a) the harm/probability ratio of given genetic variants found in probands, and b) the actionability of these variants, i.e. opportunities for (medical and or reproductive) prevention.

Secondly, distributive justice regards the just allocation of scarce financial resources and manpower for health care. Taking account of the opportunity costs of CT, priorities have to be set, at least in collectively funded or solidarity-based healthcare systems. Likewise, given limited manpower, allocating scarce medical personnel (e.g. genetics health professionals) for a wider implementation of CT may result in longer waiting lists for other healthcare services which may become overburdened.

These justice-linked, broader implications of CT may be subsumed under the heading of proportionality *in a broader sense*. Whatever the labelling and structuring of the different issues, it should be noted that considerations of both proportionality in the strict sense and distributive justice support CT focusing on cases with a higher risk of serious, avoidable harm (e.g. in the fields of hereditary cancers or inherited cardiomyopathies and arrhythmias).

## Toward a more supportive and active approach in cascade testing: removing barriers

There is a strong consensus in clinical genetics that the proportionality of CT is most obvious in the case of a *high* risk of *serious harm that can be avoided or substantially reduced*. HBOC, Lynch syndrome, and MEN type 2A are paradigmatic cases. The penetrance of the relevant genetic variants is very high, the disorders are serious, and they meet the criterion of (evidence-based) medical actionability. Still, in many countries, CT is under-used even in these more clear-cut cases.

### Barriers and facilitators

Systematic reviews [9, 12] identified the individual, interpersonal and environmental barriers and facilitators of CT aimed at optimising its effective implementation.

Individual barriers are diverse, including lack of knowledge among probands and/or relatives, discomfort with the topic, difficulty sharing information, anxiety and guilt, privacy concerns, fear of discrimination, family reaction concerns or ‘losing face’ in certain cultures. Interpersonal barriers include emotional distance, estrangement, conflict, or resentment. In contrast, environmental or structural barriers include difficulties in accessing services, limitation of services or availability of medical follow-up, and lack of insurance coverage for genetic testing, often linked with the demographic variable of having a low income [9, 12].

Individual facilitators include a personal history of probands and/or relatives with the disease [53], the receipt of an unambiguous genetic test result, knowledge of counselling or testing and risk reduction recommendations, and a (felt) moral obligation toward relatives in the form of a duty to prevent medical harm or respect relatives’ right to know’. Interpersonal facilitators are relatives being children, having a positive attitude towards disclosure, having assistance in identifying relatives and developing a plan for dissemination, and the provision of materials to pass to relatives [9, 12].

Though the authors of the reviews—Roberts and Srinivasan—rightly state that there is a need to verify the role of the barriers and facilitators identified in the uptake of CT using rigorous methods, some lessons can be distilled from the wider scientific literature and clinical experience, aimed at optimising the implementation of effective CT [54].

The focus here is on removing or mitigating individual and interpersonal barriers, as this is, at least to some extent, under the control and part of the professional responsibility of the health care professional. Regarding environmental barriers, such as possible discrimination, we stress the role of the ESHG and other relevant national genetics societies to advocate for the interests of affected families.

### A more supportive and active approach

ESHG’s central message is that a more supportive and proactive CT approach is warranted in the more clear-cut cases enabling relatives at high risk to make an informed decision about contacting genetic services and considering the possibility of testing (see below). Four aspects may be distinguished:

#### Firstly, professionals should *provide optimal support for the proband who is expected to contact relatives and is willing to do so*

Contacting and informing relatives may be difficult and burdensome for the proband, for different reasons. Helping the proband to handle this challenging task is part of providing good care. Supporting probands is especially important as non-disclosure is more likely when patients have difficulty communicating the information to their relatives, rather than being unwilling to do so [55]. The provision of support and assistance minimally includes:

- the offer of help in identifying at-risk relatives who may benefit from timely information;
- creating a dissemination plan, helping probands devise ways to better inform relatives. This may include exploring preferred communication channels between the proband and the relevant at-risk relatives and then help in building a comprehensive plan to support that communication, also taking account of the (health) literacy of the proband and relatives;
- providing an information letter to pass on to relatives when seen as helpful in the dissemination plan [56];
- the use of patient peer support [57] as well as websites and mobile apps aimed to facilitate communication in families [58–60] may have added value, though Information and Communications Technology-based solutions are less helpful for less tech-savvy people;
- offering psychosocial support;
- offering a follow-up to check whether the information has indeed been communicated to the relatives and if not, to offer additional support aimed at removing any barrier. The timing and the content of this follow-up should be discussed and clarified with the proband beforehand.

#### Secondly, modify the ethos of absolute non-directivity

Patients are usually keen to share important information with relatives, and an explicit refusal or active nondisclosure is rare [38]. However, failure of communication or passive nondisclosure may occur for many reasons. Professionals may, then, face a dilemma involving balancing the patient’s autonomy/privacy and avoiding potential harm to relatives [4, 61]. In this context, how healthcare professionals manage non-directivity in their interactions with patients may be an additional barrier to disclosing information to relatives. The principle of non-directivity is paramount in the realm of reproductive genetic counselling, especially regarding emotionally charged topics such as prenatal testing and potential selective termination of pregnancy. However, when counselling in case of a high risk for disorders for which effective and acceptable interventions are available to avoid harm, more directivity seems warranted, e.g., by stressing potential benefits of testing and making clear recommendations about passing on information. Similarly, it may be appropriate to critically examine the counselee’s preferences and values when they directly impact relatives’ health and reproductive interests [37, 41, 62, 63]. Rational persuasion to inform relatives should not be construed as violating the patient’s autonomy—it may well serve to enable a well-considered decision. Likewise, a moral appeal to parental responsibility is warranted if the proband, or a relative contacted, would be unwilling to inform his/her child(ren) at high risk of a serious but avoidable condition and/or to consent to indicated pre-symptomatic testing if the children are not yet able to give consent themselves.

#### Thirdly, accept the increasingly shared view that the importance of respect for medical confidentiality is not necessarily absolute

In the ‘high risk of serious but avoidable harm’ cases addressed in this section, relatives have a moral right to know that they are at risk. It may be possible to contact and inform relatives without violating confidentiality by giving just general information about familial risk (see section ‘The moral framework of cascade testing’). Often, however, the identity of the proband may then easily be inferred, so it is not clear if this is a solution to avoiding a breach of confidentiality. ESHG endorses the general criteria for justifiably overruling medical confidentiality, (see section ‘The moral framework of cascade testing’), affirming that:

- Anonymity should be preserved wherever possible.
- Overruling confidentiality can only be justified in the case of a so-called ‘conflict of duties’.
- In complex situations clinicians should seek consensus among colleagues, seek advice from an ethical committee, or other arrangements in their institutions rather than taking individual decisions.

If the relative at-risk is, like the proband, a patient of the HC professional involved, the situation may be different in that, then, the fiduciary duty towards this relative-patient prevails, especially if he/she asks the caregiver for information/advice.

Professionals should always account for the jurisdiction in their own country, as some jurisdictions may take a stricter view about medical confidentiality, while other jurisdictions view this differently and even translate the moral obligation to inform relatives into a *legal* obligation. In addition, clinical practices need to be compliant with the General Data Protection Regulation (GDPR), a topic ESHG PEC will be focussing on in an upcoming document.

#### Finally, *elements of ‘direct’ contacting (i.e. contacting by a professional) may be considered an alternative to traditional proband-mediated contacting* [10, 64], when needed

There seems to be growing support internationally for direct contact [9, 15, 33, 65, 66]. A prospective study of families with *BRCA* pathogenic variants concluded that using a direct contact protocol to notify at-risk relatives nearly doubled the number of relatives tested and was also found to be psychologically safe [67]. However, a recent randomised controlled trial on informing relatives at risk of inherited cardiac conditions that compared current practice with a tailored approach asking patients’ preference (relatives to be informed by themselves or by a genetic counsellor), reported no differences in uptake and impact on family/psychological functioning. Still, probands in the tailored group reported higher satisfaction levels [19]. Similarly, no increased uptake was reported after introducing a more proactive procedure allowing the geneticist to contact at-risk family members in case of a pathogenic *BRCA1/BRCA2* variant directly [7].

The term direct contacting may cover two different approaches:

1. The proband takes the initiative, informs the relatives in general terms that relevant, possibly useful, genetic information is available, and asks them whether they want to be ‘directly’ contacted by a professional for more specific information, and asks permission to give the address of the family members to the health care provider. In practice, not all elements listed may be neatly followed. Still, as Newson and Humphries [10] indicated, ideally, the proband should have an adequate opportunity to communicate with his or her family members before contact is initiated by the cascade screening programme or health care provider.
2. A professional takes the initiative, given the consent of the index patient, at least in principle.

Depending on the relevant jurisdictions, in some countries, such direct contacting approaches, supported by the ESHG, could be further operationalised. A recent guideline in the Netherlands recommends that direct contact should always be *offered* to support the index person [21]. If follow-up shows that informing relatives by the index person did not occur, it should be discussed whether direct contact is the way to proceed. This may involve obtaining addresses from the central administration, although this assumes that the proband has provided enough information for this step to be feasible in practice, and will not be allowed in some countries. In France, since 2011 (updated 2023), a procedure has been introduced by law to encourage, first, the index person to communicate directly with his/her family members by means of an information document prepared by the physician and, second, if the patient does not wish to communicate the information, the physician can directly communicate with the family members through an anonymous procedure providing only general information [68].

Busy genetic professionals may find this approach too time-consuming and there may be different solutions to tackle this problem, depending on the context and relevant jurisdiction. One option could be to include (genetic) nurses or genetic counsellors in the more routine parts of the cascade testing. Alternatively, for some disorders a public health approach using centralised cascade screening conducted by health institutes could help [12].

Increasingly, digital communication is becoming widely available and the use of health portals or mobile applications could be explored to improve information provision [59, 69].

## More complex cases require a more cautious approach

While the former section regards clear-cut cases, characterised by a *high risk of serious, avoidable* harm (also given the availability and acceptability of interventions) for relatives, this section focuses on scenarios where the possible (medical and/or reproductive) benefits of CT for relatives may be less evident and/or where the possible risks and burdens for relatives may be larger, possibly affecting the proportionality of CT.

More specifically, this section’s focus is on three types of scenarios that differ in at least one relevant aspect from the clear-cut cases:

A. The genetic risk for relatives is moderate to low.
B. The actionability is limited:
  1. The medical actionability is very limited;
  2. The genetic risk is potentially actionable in reproductive terms, but non-actionable
  (not preventable/non-treatable) in medical terms.
C. The condition is mild.

How do these modalities impact the practice and morality—the ‘If and How’—of CT? Is CT still warranted/proportional in such cases, and should some additional conditions and safeguards be considered? Is it appropriate to recommend a more active approach here, in line with the clear-cut cases addressed in the former section, or is it wise to limit this more active approach to these clear-cut cases?

It will be argued that in the scenarios of more complex cases, a more cautious approach is relevant as the balance between the benefits and harms of CT is less obvious. This more cautious approach does not call for directivity, the relaxing of medical confidentiality or for direct contact. During counselling, it should be assessed whether family members should be informed of their risk. If it is decided to inform the family, the focus should be on counselling and provision of information, rather than on offering the test itself, for which the term ‘cascade counselling’ would be more appropriate.

While certain hypothetical CT cases may appear proportional in a narrow sense, where benefits for relatives seemingly weigh more than the consequences for the individual, these may still be disproportionate when considering the broader societal consequences of CT. Given resource and workforce constraints, some CT cases might not be prioritised by clinical genetics centres or insurers.

The reflections above apply to (likely) pathogenic variants. In relation to VUSs, we recognise that while in selected cases inheritance studies might aid interpretation of the variant for the benefit of the proband, this would not be considered a properly defined CT, in that there would be no clear benefit for the relatives. CT for VUSs would not be recommended due to potential harm arising in the case of a misinterpretation of the result.

### Moderate to low risks

Below are two examples: [1] moderate to low cancer risks: lower-penetrance Mendelian cancer predispositions and low-risk cancer susceptibility genes; and [2] low-risk carrier status of an AR condition. What about the ‘If and How’ of CT in these cases?

#### Moderate to low cancer risk

A good example regards breast cancer. While there is a strong consensus that breast cancer genes can be categorised as high-, moderate- or low-risk, experts have somewhat different views about the demarcation, and classification may be different when these genes are assessed for other types of cancer.

Examples of the different risk categories for breast cancer are:

- *High* risk (relative risk—RR—≥ 4X general population risk): such as *BRCA1, BRCA2, PALB2*;
- *Moderate* (or *intermediate*) risk linked to a Mendelian condition (RR ≥ 2 < 4): such as *ATM* and *CHEK2*;
- *Low risk:* this includes the numerous genes involved in the common multifactorial cancers; they are usually associated with very low increases in risk compared to the general population.

While the offer of more active CT will be appropriate when it concerns high-risk Mendelian breast cancer predispositions, the urgency of CT is lower in the case of moderate-risk factors and even less in the context of low-risk susceptibilities. Although the latter ones are not useful individually, they could be considered collectively in the frame of polygenic risk scores for risk stratification and/or for a better estimate of the variable risks associated with high and moderate penetrance genes. However, these potential applications are not relevant for CT. Also, proportionality in the wider sense may be a limiting factor, as a shortage of resources and personnel may lead genetic services to set priorities and focus on high(-er) risks.

Whatever the outcome of such priority setting, the more active approach recommended above for clear-cut CT cases seems less warranted in cases of moderate to low risks. More specifically, when the benefits to be gained from CT are less clear, health professionals will be less justified in making frank recommendations about the need to pass information to relatives or in breaking medical confidentiality. A more traditional approach to such non-directive counselling regarding informing the family and respect for confidentiality would be appropriate. Of course, this discussion has been framed in terms of ‘objective’ (evidence-based) levels of risk. The perspective on the level of risk as perceived by a patient may differ substantially from how their risk is evaluated by health care practitioners. In such a situation, the discussion must work with the patient, starting from their situation as they perceive it, while aiming to ensure that they understand the medical results and their implications. Although it is possible that health professionals and information recipients may have different understandings and are unable to achieve an agreed understanding, it is important that professionals should not collude with misunderstandings or agree to act on the basis of incorrect understandings and hold in mind ‘first do no harm’.

#### Carrier status linked with AR inheritance

In the case of AR inheritance of a disorder manifest in the proband or AR carrier status, the relatives’ repro-genetic risk also depends on the carrier status of the partner. For the vast majority of AR conditions, heterozygous carriers are not at (significant) risk themselves of developing a disease, but they might have an increased reproductive risk for the condition. This is of course conditional to their reproductive partner also being carrier of the same AR condition. In this scenario, the utility of CT in the family intersects the opportunity and availability of carrier screening for their unrelated partners. CT aimed at identifying carriers (carrier couples) of AR disorders among relatives is widely considered to be proportional on conditions, for instance:

a) there is a degree of consanguinity, or

b) the carrier frequency of known pathogenic variants in the relevant population is high (for example, at least 1:100, e.g., as recommended in the United Kingdom 1:70) [70], and therefore the carrier screening in the partner would be offered and

c) the relevant disorder is serious (see below), and reproductive (preimplantation genetic testing (PGT), prenatal diagnosis) and/or management options would be available.

A proactive CT approach in this scenario would seem therefore proportional to the level of actual risk and the available options for actionability—including here actionable reproductive options. However, in the setting of reproductive decision making it is always important to take into consideration that women may reject the offer of having their partner tested if they are aware there is a risk that the ‘social father’ is not the ‘biological father’ [71, 72]. In taking an active role to ensure fathers are tested, there is the risk for psychosocial harm for the family unit if such testing triggers separations or aggression.

### Partial or limited actionability

We distinguish two types of cases:

#### Limited medical actionability: the case of Li-Fraumeni syndrome (LFS)

Long-standing hesitancy to offer pre-symptomatic genetic testing for LFS is due to ineffective surveillance, as carriers are at high risk of developing cancer everywhere in the body. More recent studies, however, suggest that promising surveillance strategies have become available. Against this background, various expert groups, including the European Reference Network for Genetic Tumour Risk Syndromes (ERN GENTURIS), a panel of the American Association for Cancer Research and a UK Cancer Genetics Consensus Group (UKCGG), recommended that patients with LFS be offered cancer surveillance using a modified version of the so-called Toronto protocol that includes whole body-magnetic resonance imaging [73–76].

In light of the above, CT may be offered for LFS, though, for the time being, caution is warranted for various reasons. First, the UKCGG rightly stipulates the lack of robust evidence to base their discussions and recommendations on [74]. Secondly, healthcare professionals have to be aware of the huge psychological impact of being a carrier of LFS, particularly given its limited actionability [77].

Both factors impact the ‘how’ of CT for LFS. Though pre- and post-test genetic counselling should always be part of predictive genetic testing, this is especially important in less straightforward cases, like LFS. The UKCGG rightly recommends that discussing the screening recommendations with patients should include a thorough discussion of the *pros* and *cons*. This is not just a cognitive issue: as important is the counselling regarding the ability of patients to handle the burdens of knowing about their LFS-carrier status and the uncertainties inherent in the recommended surveillance in LFS carriers. The reproductive implications of LFS and the options for ‘avoidance’ of transmission (incl. PGT for monogenic disorders; PGT-M) should also be addressed in the genetic counselling.

Ideally, CT for LFS should be embedded in scientific research, both medical and psychological. ESHG endorses the view of the UKCGG, which recommends that screening data be prospectively collected and audited to inform future practice and fill gaps in the understanding. Psychological research is needed to better understand the needs of families with and individuals at risk for LFS. Scientific research is especially important when CT involves children, also as no established surveillance exists for many paediatric cancers [78].

Given both the medical uncertainties and the possible psychological burdens of presymptomatic testing for LFS, the focus should be on cascade *counselling* (CC) about disclosing information to family members. A more directive style of counselling—making unambiguous recommendations for the proband to inform family members—seems to be unwarranted. Likewise, it seems to be difficult to justify overruling confidentiality at present as the burden of being identified as a carrier is high and the benefits of intervention are unclear.

#### Reproductive actionability: neurogenetic conditions—the case of Huntington Disease (HD)

What is typical for the Huntington-case and similar neuro-genetic cases, like early-onset dementias and the dominant spinocerebellar ataxias, is that the relevant risk information is so far non-actionable in medical terms, but at the same time actionable in terms of planning life and in reproductive terms. Relatives at risk may want the information to make an informed choice, including the making of life plans and reproductive options such as PGT-M [79]. How does this impact the proportionality, the ‘If and How’ of CT for HD?

In this setting, we must be clear that in the case of HD, the idea of Cascade *Testing* as the default is inappropriate. When considering CT for untreatable conditions, we are on different ground, also because of the grave psychological impact on a proband and family members when learning about such a disorder in their family [80]. Therefore, what is a serious consideration is whether, when and how to pass information to relatives, i.e. cascading *information* or cascade *counselling*. Indeed, when an at-risk family member has only just found out about their risk of HD, and similar conditions, it is important that the discussions about testing are seen as an ongoing discussion which is patient-led. It is also good clinical practice to counsel a proband to be open about the risk of HD in a family and advise careful consideration about the negative impact of secrets on family members. The need to foster and encourage open family communication needs to be balanced with the acknowledgement that family communication on this topic is challenging, and that it is not uncommon for families to hold secrets [81]. Some authors oppose the notification of relatives on grounds of their right to ‘not know’ about being a member of an HD family [82, 83] (see section ‘The moral framework of cascade testing’). The alternative view, however, widely supported in clinical genetics, is that CC for HD may well be responsibly implemented, for different reasons. First: in many cases, relatives will find out about their genetic risk in unplanned and upsetting ways, for example, by a relative becoming symptomatic. The discovery that they were not told earlier may result in serious intra-familial conflicts [84, 85]. A second reason that has gained some recent support is that CC, followed later by the offer of presymptomatic testing, may help some relatives prepare for the future and make relevant life choices. For example, carriers may choose to engage in environmental stimulation (adopt an active lifestyle, behavioural therapies, diets, etc.) that might positively influence HD’s onset (Rhona MacLeod, *pers. comm*) [86]. Thirdly, CC addresses relatives’ reproductive interests; not informing them will remove their ability to make such a choice.

While ESHG agrees that CC for HD may be good clinical practice, some comments on these reasons in favour are warranted. Most importantly, though the reproductive benefits for relatives may be real, they are not self-evident. Firstly, it is often not known whether relatives want to have children in the future. And, secondly, the assumption that relatives who want to have children while being informed about their high a priori risk usually engage in pre-symptomatic testing and, if proven to be a carrier, take preventive reproductive measures is incorrect. Only a small minority (10–20% in many countries) of relatives at 50% risk for HD (and similar disorders) have pre-symptomatic testing [87]. Furthermore, clinical experience and research show that many people at high risk choose to have children without any genetic interventions, as has been found in other conditions [88, 89]. Regarding HD this has occurred for various reasons, including the hope that preventive therapies for severe untreatable conditions will become available in time [88, 89]. Other research suggests that couples may make rather different reproductive choices related to subsequent pregnancies (‘after the test, choices recommence’) [90]. The place of reproductive technologies, while worth consideration, should not be exaggerated. PGT-*exclusion* testing, enabling selective reproduction while circumventing pre-symptomatic testing in people at 50% risk who prefer not to know their possible carrier status, has an added value for a considerable number of prospective parents at risk for HD [79]. However, this is not a real option for many people because such testing is not allowed in their country and/or will not be reimbursed. Finally, PGT is a burdensome process; couples often change their minds and refrain from reproductive assistance after their first attempts [91].

An important factor to keep in mind, and that sets the tone for this section, is that people can understandably feel anger when they discover important information has been deliberately withheld from them by others, whether relatives or healthcare professionals. This can lead to serious family rifts and stressors. Again, we urge a compassionate approach to this, as it is not uncommon for people to hold secrets from their family members based on a belief that they are protecting them or themselves [92]. An approach that helps them carefully balance the impact of information disclosure/non-disclosure is likely to be most helpful in ensuring families share information and adapt to it. This shapes our view on the importance of promoting CC rather than CT for HD and similar conditions. However, although loath to break respect for patient confidentiality in these cases, there will be times when it is ethical and appropriate or even (in some jurisdictions) legally required.

### Less straightforward, including less serious, disorders

There is strong support for CT if this informs relatives about the risk of developing and/or transmitting an avoidable *serious* disorder. As we all know, ‘serious’ is notoriously difficult to define [93]. One example could be non-syndromic hearing impairment (HI). HI is not life-threatening, nor reduces expected lifespan—does this qualify for a less serious handicap? The inheritance pattern of HI differs; autosomal recessive (in about 77% of the hereditary cases), autosomal dominant (in some 22%), and X-linked, or mitochondrial (in the remaining 1%) [94, 95]. It is important to distinguish between severe HI (i.e. deafness), which is most often autosomal recessive and congenital, and autosomal dominant HI, which is often mild to moderate and sometimes progressive, because the impact on the affected families may differ considerably.

HI is especially interesting as it is linked to the debate about the social versus the medical model of disability [96]. While the medical model considers a disorder primarily as a medico-biological problem, the social model considers a handicap to arise out of the interplay with the socio-cultural environment. Avoidance of HI using PGT or prenatal diagnosis may, from the latter perspective, be considered to be both a symptom and catalyst of a contested medicalization of HI - the real problem is societal, namely the stigmatisation/exclusion of and societal barriers to the participation of people who are ‘different’. However, some prospective parents may feel HI to be a significant handicap, to be avoided for future children.

This is one example where the view of how serious the condition or its impact is will vary considerably between people. This in turn will impact their views about the desirability of CT and using it to make reproductive decisions [93]. Of note is that these views can vary between partners, and between parents and their wider families, again leading to a suggestion for the need for CC, rather than just CT.

While ESHG recommends further debate and reflection, for the time being, it takes a pragmatic and consensual view: what constitutes a risk of a ‘sufficiently serious’ condition, as a starting point for CT, will usually be agreed upon via multi-disciplinary debate, taking account of the voices of patients/patient organisations and experienced clinicians. Requests for prenatal testing and PGT may, then, be valuable indicators of how seriously to regard this condition [97, 98]. No doubt, HI is less serious than many other genetic disorders, but still, HI in many cases is not minor, let alone trivial. In the case of both AR HI (the large majority of early-onset severe cases, where relatives’ risk of having an affected child is low) and AD HI (a higher risk, but a mostly mild phenotype), a combination of arguments makes the proportionality of CT debatable, both in the strict sense and in the wider sense, taking account of the scarcity of financial and human resources.

Newborn hearing screening for severe AR HI, representing a functional identification of HI of a degree requiring treatment with hearing aids and/or cochlear implants, is widely implemented, and this has resulted in substantial improvements to quality of life and the opportunities for communication in those affected.

Most cases of AD HI are not so severe that the term deafness should be used. Most cases are milder/moderate and sometimes progressive. In AD cases, most families recognise the HI as a familial characteristic and will seek audiological evaluation in due time.

In any case, given both the less straightforward character of HI and the fact that neonatal screening will enable early detection of moderate/severe HI and adequate treatment of congenitally affected children, the active approach, as recommended for the clear-cut cases, is unsound also in this case: directive counselling and the overruling of confidentiality would usually seem to be inappropriate and unjustified.

## Cascade testing beyond the clinical genetic practice

### Mainstreaming genetic testing: opportunities and challenges for cascade testing

Traditionally, in rare disorders, clinical geneticists and genetic counsellors have discussed the relevance of a diagnosis for the family with their patients. In recent years, for some disorders, diagnosis and, to a lesser extent, informing the family, are becoming integrated into mainstream care. Various scenarios have emerged in which genetic and non-genetic healthcare professionals intensify collaborations, sometimes organising care in multidisciplinary teams or subfields, such as oncogenetics and cardiogenetics. In cardiogenetic disorders, genetic testing and CT could be the responsibility of the cardiologist or a cardiogenetic specialist, as this may involve, e.g., imaging and/or electrocardiography to assess which family members are at risk. For instance, in hypertrophic cardiomyopathy, according to recent guidelines [99] screening first-degree family members can be performed by using either genetic testing or imaging/electrocardiographic surveillance. In oncology, in some countries, after mainstream testing for *BRCA*-related breast and ovarian cancer, when a positive test result is obtained, patients will be referred to the clinical geneticist who will be responsible for counselling and possibly cascade testing [100].

In FH, diagnosis will often be made in primary or secondary care via cholesterol measurement or after the occurrence of cardiovascular problems. Requesting genetic testing and discussing the test results and their relevance for family members in some countries may also be done by the primary care physician or non-genetics health care professionals, often in internal medicine.

Challenges for CT to be offered in mainstream care may relate to practical skills in drawing pedigrees and the selection of family members to inform, but also to counselling skills and ways to support the proband in responsibly informing their family members. Clinical geneticists have an important role in training and coaching non-genetics healthcare professionals and being available for expert advice and follow-up [101].

When a condition can only be diagnosed clinically, and no genetic cause can be identified, the emphasis will be on cascade *counselling* instead of testing. A close collaboration with the genetic service will facilitate communication within the family, promote proactive health monitoring, and encourage the re-analysis of genetic data to identify potential new disease-associated genes.

Considering the active approach outlined in the previous chapters, a particular challenge relates to ascertaining for which disorders and contexts a more active role for the mainstream professional towards CT is warranted, e.g. by more directively recommending the sharing of relevant risk information with first-degree relatives to start with.

### Cascade testing and public health

CT should be more directively encouraged in the ‘clear-cut’ cases when it is possible to avoid serious harm by informing family members of their increased risk of disease, and an acceptable intervention is available. However, such an active approach to CT would consume resources and impose a substantial burden of opportunity costs. Especially for cases such as FH, *BRCA*-related breast cancer and Lynch-syndrome-related cancers, mainstreaming can be helpful in the rational use of resources, allowing geneticists to focus on more difficult cases or the families of confirmed cases. Other than mainstreaming, more structural solutions have been developed for some disorders to allow more systematic case finding and informing family members. CT may be supported by a specific organisation or professional role, allowing efficient resource use and optimal information provision. In Norway, since the early nineteen nineties, in case of FH a dedicated counsellor contacts family members after an index patient has asked them if they want to be contacted by the healthcare system [102]. After counselling, the relatives can contact their GP for genetic testing. In the Netherlands, a foundation was created to support a national FH screening programme (1994–2013). Index patients would ask relatives if they agreed to be contacted. Genetic field workers would visit family members at home to inform them and draw blood. Familial mutations were recorded with family names in a registry, thus new patients of known families could be tested relatively cheaply for the familial mutation. Family tracing was covered via public health funding. This programme was cost-effective and accepted by patients [103], but it was assumed that after 20 years, most families would have been detected [104]. After the programme ended, more local solutions were found, and in some centres, a dedicated nurse was appointed to discuss and support informing the family. However, the number of family members tested dropped considerably nationwide [11, 104]. A recent paper discussing Dutch and Norwegian experiences suggested that CT for FH should be organised nationally [105] to increase efficiency. Similarly, Roberts et al.’s review [12] of CT delivery suggested that a public health approach using centralised cascade screening conducted by health institutes could help facilitate providers contacting at-risk relatives. Whether this is feasible varies across national contexts and jurisdictions. Furthermore, FH characteristics (high carrier frequency, an acceptable intervention that can be administered and monitored in mainstream care) may enable a public health strategy. In Denmark, a national screening programme to identify mismatch-repair defects has been introduced for newly diagnosed colorectal cancer. After 10 years, a high uptake was reported, although disparities in access to testing and suboptimal referral to genetic counselling in individuals suspected of Lynch syndrome were documented [106]. Whether such screening strategies would work for other disorders needs to be explored.

Another strategy to optimise CT—but potentially further increasing workload in medical specialties involved—would be to combine strategies and put more effort into follow-up of cases identified via population screening with counselling and CT. In a review on FH, CT of family members after a population screening in children has been proposed as an effective means to identify individuals at risk [107], although this will not be an effective way of identifying those affected adults without children or those with children to whom they have not transmitted the genetic disorder.

A more widely discussed route is CT after newborn screening (NBS). NBS in many countries will identify newborns with autosomal recessive conditions and, depending on the country and the technologies used, sometimes carriers. It has been argued that reporting carrier results would allow parents to opt for genetic testing to establish their risk of having an affected child in a subsequent pregnancy [108, 109]. This would be especially relevant for countries that do not have adult carrier screening programmes available. However, parents may not act upon the information for subsequent pregnancies, as studies have indicated for sickle cell disease (SCD), [110, 111]. Research has shown that parents value CT for their other children as enabling closure following carrier identification via NBS. However, they also reported struggling to access CT due to unclear pathways [112]. Although it is argued that as NBS has been running for sufficient years so, parents will know whether any of their children are carriers of disorders for which carrier status is reported, the situation is more complex. Parents move between countries with different conditions included in the screening and different reporting protocols. Although the Borry et al. [39] guidelines can be upheld to offer testing at a later age, facilitating discussions on risk levels for young adults and children post-NBS within families, and elucidating known factors, may be beneficial. Even when it is known by professionals and their parents that a child was found to be a carrier, whether this becomes known to the child is not straightforward [92], further strengthening the calls for support for families to have these conversations. Empirical work is ongoing to map and evaluate such experiences, which also uncovers considerable variation between conditions, with parents accessing carrier testing for their children for SCD more readily [71, 113].

### Opportunistic genomic screening in cascade testing

If the offer of testing to blood relatives would also include genetic variants unrelated to the original health problem found in the proband, CT would transform into opportunistic genomic screening (OGS), defined as an unsolicited offer of a test without an indication for this test. In a former document, ESHG addressed OGS, arguing that it should meet the criteria of proportionality, respect for autonomy and justice [23].

Firstly, proportionality requires that the benefits for those screened outweigh the possible harms. Given the many unknowns, any OGS in CT would need to be embedded in pilot and evaluation studies. Secondly, regarding autonomy, a major challenge is how best to guarantee ‘truly informed’ decision-making. And thirdly, OGS raises issues linked to justice. Concerning the additional OGS, relatives initially offered targeted CT for the specific variant found in the proband do not have a higher a priori risk than other members of the general population. Such inequality of access to screening may be difficult to justify. The suggestion to avoid this problem by a universal screening offer (‘for all’) raises issues linked with the just distribution of resources for health care. Given the scarcity of both resources and qualified man-power stringent criteria will be needed for any systematic broader sequencing [23].

Given the concerns, some possible conditions regarding OGS in CT may be imposed: firstly, the implementation of a multi-step, dynamic counselling and consent process [114] aimed at separately addressing possible secondary findings, and secondly, avoiding offering CT for rather different types of conditions and (medical or reproductive) risk information simultaneously.

Conversely, CT could be offered to blood relatives of a carrier identified via OGS or other forms of screening or testing leading to secondary or incidental findings. Finding a pathogenic variant would warrant CT, although caution is necessary, as penetrance in families without a family history for the related disorder may be reduced [25].

## Recommendations

Cascade testing (CT) involves a systematic approach to identifying relevant family members to inform and counsel them about their risk of developing the disease already identified in a relative or having a child affected by that disease, and then to offer them targeted genetic testing for that genetic disorder. In some circumstances, there will be a greater emphasis on information provision and counselling as an initial step, which is termed cascade counselling (CC). This process is repeated in subsequent degrees of kinship as more carriers are identified in the family. Cascade counselling and testing is an effective instrument to find and inform individuals at risk, enabling informed decision-making primarily on preventive interventions and reproductive options.

### Weighing benefits and harms

CC/CT harbours an element of screening as it involves an unsolicited offer to family members who often will not be aware of their higher risk, nor have experienced health problems related to that genetic disorder.

CC/CT can be justified as a proportional intervention if the potential benefits for the relatives outweigh the possible harms. Whether CC/CT meets this proportionality requirement depends on the context. We distinguish three scenarios relevant for deciding *whether*, and, if so, *how* to offer cascade testing. In more clear-cut cases, a more active approach is warranted, while in complex cases, a more cautious approach should prevail with a focus on CC. In some cases, CT may not be proportional or appropriate.

### An active approach

The proportionality of CT is most evident in the case of a high risk of serious, avoidable harm or suffering. Examples would include highly penetrant pathogenic variants where acceptable courses of action, such as surveillance, preventive interventions, options to avoid an affected pregnancy, and follow-up care, are possible. In such cases, it is reasonable to anticipate that relatives will significantly benefit from receiving this information. A shared moral duty to inform relatives at risk may be identified as an obligation on the part of first, the proband and second, the health care professional involved, though this will depend on national regulation and GDPR requirements. In this subset of cases, an active approach to the cascading of information, counselling and testing for family members can be recommended. This may include:

- A more directive approach, aimed at persuading the proband to inform family members;
- Active support for the proband in taking steps to inform the family, e.g., discussing a dissemination plan, including distributing a family letter if this would be helpful as discussed with the proband, and checking with the proband if they were able to pass the information;
- Offering to contact family members directly, ideally, after the proband has asked family members for permission to be contacted, depending on national regulations;
- A less stringent approach to medical confidentiality in informing family members in case of a high risk of serious avoidable harm, and the proband is unwilling or incapable of informing the family, depending on national regulations.

### A more cautious approach

A less obvious balance between the possible benefits and harms of CT warrants a more cautious approach, which should be assessed during counselling. Caution is warranted especially when health risks are less obvious or less serious, penetrance is low, no medical treatment is available, or actionability is limited. This more cautious approach does not call for directivity, nor for the compromising of medical confidentiality or for direct contact. During counselling, it should be assessed whether family members should be informed of their risk. If it is decided to inform the family, the focus should be on counselling and provision of information, rather than on offering the test itself, for which the term ‘cascade counselling’ could be appropriate.

### When cascade testing is not proportional or appropriate

In some cases, CT may not be proportional or appropriate given the debatable balance between benefits and harms. The assessment of these situations should also be seen in relation to available resources. Relevant cases include: 1. In autosomal dominant disorders where the penetrance is low, and the risk of developing the condition is only slightly higher than in the general population;

2. In autosomal recessive disorders which are ultra-rare in that population, so that carrier testing of the partner would not be offered. 3. CT for VUSs is not considered appropriate as there would be no benefit, and potential harm in case of misinterpretation.

### Counselling

Counselling should always be part of CT and is to be provided by a qualified professional, such as a genetic counsellor, clinical geneticist, genetic nurse, or appropriately trained healthcare provider. When informing family members is a part of a specialist trajectory in e.g. oncology or cardiology, genetic expertise should be available within a multidisciplinary team.

Counselling should promote patient empowerment based on an active deliberative process. If index patients are willing to inform relatives themselves, healthcare professionals should assist in identifying at-risk relatives and co-creating a dissemination plan that considers timing and content of communication, including handing out appropriate written materials to pass on to relatives. Alternative and supplementary ways to inform relatives should be discussed. Follow-up contact should be tailored to patients’ preferences and service availability. Awareness of the type and scope of tests used by the laboratory is crucial to allow for adequate counselling, also regarding possible incidental or secondary findings. In such cases, a multi-step, dynamic counselling and consent process is preferred.

### Removing barriers

Research should be promoted on identifying and removing barriers to familial communication, supporting probands and accessing genetic services. It may be efficient to use public health facilities and involve genetic counsellors, nurses, and patient organisations. Using modern communication channels such as online tools and health portals is important, without losing more personal communication options for people who are less able to use modern media or mobile applications. In some countries, registries can facilitate familial testing, monitoring and research.

### Minors

Minors’ (future) health interests as well as their (future) autonomy and privacy interests should be considered when assessing whether CT is justified. When an intervention, including surveillance, is recommended and available to prevent imminent serious medical harm to the minor, a more directive counselling of parents may be justified. If, however, genetic risk regards a later-onset disorder cascade testing, in principle, needs to be postponed until the grown-up child has decision-making capacity. Cascade counselling may be an efficient way to inform families about familial risk in case the offer of testing children is not appropriate yet.

### Resources

Considering the inevitably limited available resources, including healthcare budget and staff, the proportionality of CT should also be assessed to make the process sustainable in the long term for those who will benefit from it the most.

### Evaluation

As research continues into the effectiveness and psychological impact of cascade testing, evaluation studies will be necessary to help update these recommendations and assess their acceptability and impact across different population subgroups.

## Data Availability

The preliminary version and anonymized comments made before the final version are available from the first author upon request.
